# Model of Tooth Morphogenesis Predicts Carabelli Cusp Expression, Size, and Symmetry in Humans

**DOI:** 10.1371/journal.pone.0011844

**Published:** 2010-07-29

**Authors:** John P. Hunter, Debbie Guatelli-Steinberg, Theresia C. Weston, Ryan Durner, Tracy K. Betsinger

**Affiliations:** 1 Department of Evolution, Ecology and Organismal Biology and School of Earth Sciences, The Ohio State University, Newark, Ohio, United States of America; 2 Department of Anthropology, The Ohio State University, Columbus, Ohio, United States of America; 3 Department of Evolution, Ecology and Organismal Biology, The Ohio State University, Columbus, Ohio, United States of America; 4 Department of Anthropology, State University of New York at Oneonta, Oneonta, United States of America; University of Illinois at Champaign-Urbana, United States of America

## Abstract

**Background:**

The patterning cascade model of tooth morphogenesis accounts for shape development through the interaction of a small number of genes. In the model, gene expression both directs development and is controlled by the shape of developing teeth. Enamel knots (zones of nonproliferating epithelium) mark the future sites of cusps. In order to form, a new enamel knot must escape the inhibitory fields surrounding other enamel knots before crown components become spatially fixed as morphogenesis ceases. Because cusp location on a fully formed tooth reflects enamel knot placement and tooth size is limited by the cessation of morphogenesis, the model predicts that cusp expression varies with intercusp spacing relative to tooth size. Although previous studies in humans have supported the model's implications, here we directly test the model's predictions for the expression, size, and symmetry of Carabelli cusp, a variation present in many human populations.

**Methodology/Principal Findings:**

In a dental cast sample of upper first molars (M1s) (187 rights, 189 lefts, and 185 antimeric pairs), we measured tooth area and intercusp distances with a Hirox digital microscope. We assessed Carabelli expression quantitatively as an area in a subsample and qualitatively using two typological schemes in the full sample. As predicted, low relative intercusp distance is associated with Carabelli expression in both right and left samples using either qualitative or quantitative measures. Furthermore, asymmetry in Carabelli area is associated with asymmetry in relative intercusp spacing.

**Conclusions/Significance:**

These findings support the model's predictions for Carabelli cusp expression both across and within individuals. By comparing right-left pairs of the same individual, our data show that small variations in developmental timing or spacing of enamel knots can influence cusp pattern independently of genotype. Our findings suggest that during evolution new cusps may first appear as a result of small changes in the spacing of enamel knots relative to crown size.

## Introduction

The Salazar-Ciudad and Jernvall [1] model of tooth morphogenesis accounts for the development of tooth shape, including the appearance of new cusps, based on a small number of developmental parameters. Initiation and placement of presumptive cusp tips, epithelial folding and mesenchymal growth, cessation of crown formation and beginning of root formation all affect tooth crown shape by determining the size and shape of the epithelium of the developing tooth that will become the enamel-dentine junction of the fully-formed tooth.

The model predicts covariation among morphological variables, such as tooth size, intercusp distances, and cusp size. The model hinges on the molecular signaling activity of enamel knots, transient structures composed of nonproliferating epithelium that direct the folding of the dental epithelium at the future sites of cusp tips. In addition to promoting epithelial and mesenchymal growth, enamel knots also produce inhibitors which prevent the formation of new enamel knots within a zone surrounding them. As the distance from a preexisting enamel knot increases, the likelihood of escaping the inhibition field surrounding that enamel knot increases, which may result in the formation of a new enamel knot, and thus a new cusp. All else being equal, a larger tooth bud or a tooth with smaller distances between the earlier forming enamel knots may be more likely to allow late-forming enamel knots to initiate around the periphery of a tooth crown. The model is applicable to all vertebrate teeth and has been used successfully to predict patterns of cusp expression in other mammalian species [2].

**Figure 1:**
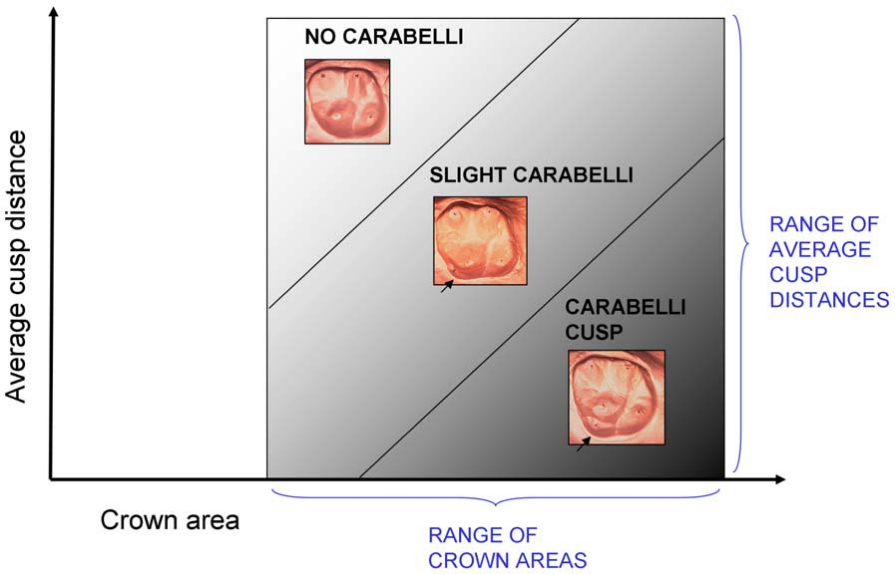
Predicted associations between cusp spacing and tooth size with Carabelli expression.

Here we test the model for its predictions about the expression, size, and symmetry of the Carabelli trait in humans. The Carabelli trait emerges from the lingual surface of the protocone (the mesiolingual cusp of upper molars) and usually begins to form after the four major cusps of the molar have initiated [3] (If the Carabelli cusp is as tall as the other cusps, which it occasionally is, then it initiates with them.) The trait ranges in expression from a shallow furrow to a cusp with a free apex which rivals the hypocone, one of the molar's four principal cusps, in size [4]. We predicted that if the patterning cascade model can explain the presence and size of the Carabelli cusp, then: (1) teeth with smaller intercusp distances relative to crown size would be more likely to possess the Carabelli cusp and, (2) teeth with the smallest intercusp distances relative to crown size would possess the largest Carabelli cusps (Figure 1).

The model is also predicated on the idea that morphology is produced not by genes alone but rather by the interaction of a gene network operating in the spatial context of the developing epithelial surface that itself contributes to the final morphological form of the crown. Given such a model in which both genes and the spatial relationships of enamel knots influence resulting morphology, the association between Carabelli expression and relative intercusp spacing should extend to teeth on opposite sides of the same individual. That is, individuals with smaller intercusp distances relative to crown size on one side of the jaw should also have larger Carabelli cusps on that side. Examining asymmetries in Carabelli cusp size within individuals offers a new opportunity to test the model's predictions when genotype is held constant.

Investigations of human population affinities based on the analysis of dental morphology routinely incorporate this trait [4], which was once thought to typify particular populations of humans [5], [6]. The Carabelli trait is also present at variable frequencies and levels of expression in Plio-Pleistocene hominin species [7], [8], [9], [10]. Given the centrality of this trait to studies of evolutionary relationships among past and present humans, an understanding of the developmental underpinnings of the Carabelli trait is useful. If the patterning cascade model of tooth morphogenesis applies to the Carabelli trait, then a high potential for homoplasy in Carabelli expression as well as associations between Carabelli and other dental features can be expected [11].

## Results

We assessed error associated with our measurement protocol in a subsample of 19 teeth measured four times on separate days (Table 1). We calculated a measure of relative measurement error (ME) as a percentage of the total variation among individuals and within individuals (i.e., among replicate measurements of the same individuals) partitioned through Model II ANOVA [12], [13]. Percent ME is more influential than absolute precision of measurements in determining statistical power. ME is moderately high for the linear intercusp distances (12–32%), whereas ME is somewhat lower for tooth area (10%) and Carabelli area (4%). Relatively high ME for the intercusp distances may be due to the small magnitude of these dimensions (∼2–9 mm on average) relative to measurement repeatability (standard error of measurement ∼0.20 mm), low variation among individuals due to the functional constraints of precise occlusion, and subjectivity in locating the position of cusp tips. Measuring areas does not suffer from the subjectivity of locating cusp tips, and ME of areas may arise from variation in orienting the teeth relative to the occlusal plane. Because error in measurement should be random, its impact on our statistical tests should be to reduce power, making it more difficult to obtain significant results (i.e., increased Type II error). Although methods exist to adjust total variance by removing an estimated proportion of within-individual variation [14], such methods may inflate the probability of obtaining a false positive result (i.e., increased Type I error). Instead, we chose to mitigate the potential impact of measurement error on power by analyzing a large sample, in our case almost two hundred individual human dentitions.

**Table 1:** Measurement error (ME) as a percentage of total among and within individual variation, derived from a Model II ANOVA [12], [13] using four repeated measurements per individual in a subsample of 19 teeth.

Measurement	MS among	MS within	s2^1^ among	ME (%)^2^	N^3^
Tooth Area	241.83	6.90	58.73	10.5	19
Carabelli Area	17.92	0.21	4.43	4.5	10
Paracone-Protocone	0.49	0.05	0.11	32.3	19
Paracone-Metacone	0.20	0.02	0.05	31.7	19
Protocone-Carabelli	0.58	0.06	0.13	32.1	10
Protocone-Metacone	1.18	0.07	0.28	20.9	19
Paracone-Hypocone	0.56	0.04	0.13	24.4	19
Protocone-Hypocone	0.95	0.06	0.22	20.9	19
Metacone-Hypocone	1.09	0.04	0.26	12.3	19

^1^Estimated as (MS _among_ – MS _within_)/4 measurements per specimen.

^2^Estimated as 100× [s2_within_/(s2_within_+s2_among_)], where s2_within_  =  MS_within_.

^3^Ten teeth in this subsample possess a measureable Carabelli cusp.

**Figure 2:**
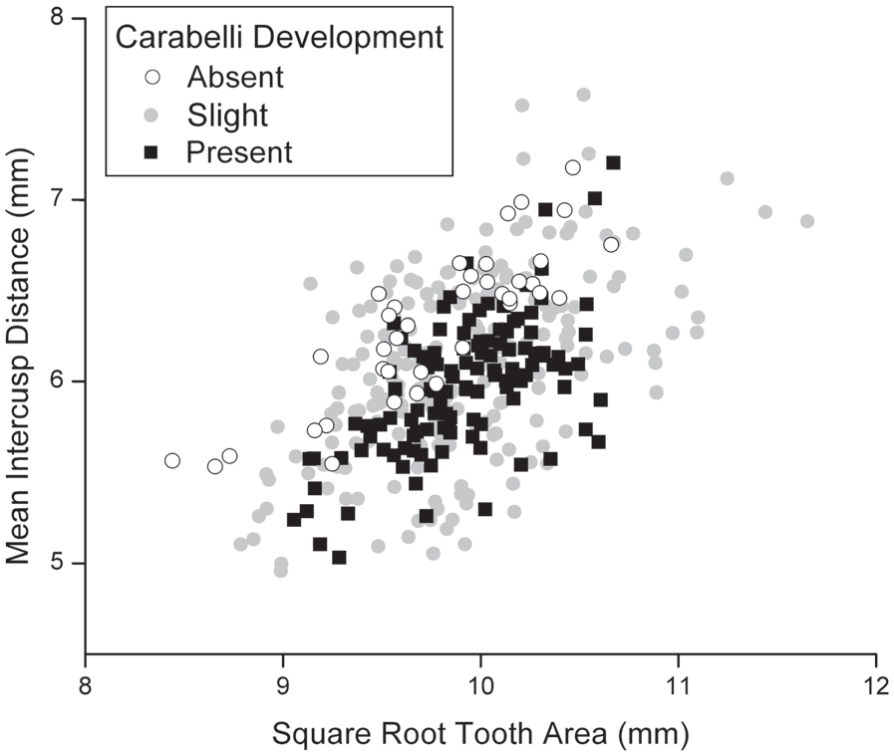
Interaction of cusp spacing and tooth size covaries with Carabelli expression.

In the full sample of 376 M1s, teeth with Carabelli cusp present clearly possess lower mean intercusp distances in absolute terms as well as relative to tooth size than teeth without Carabelli development (Figure 2). Teeth with slight Carabelli development span a range of values overlapping the ranges of the other two groups (Figure 2). For statistical analyses, we separated lefts from rights to avoid correlation between antimeric pairs. To detect any potential for autocorrelation among variables due to purely geometric concerns, we explored how mean intercusp distance and square root tooth area each separately relate to Carabelli development. For lefts, as an example, teeth with fully expressed Carabelli cusps possess significantly smaller average intercusp distances than teeth without any Carabelli development (Table 2). In contrast, teeth at the extremes of Carabelli development do not differ in our sample in square root tooth area (Table 2). Because Carabelli development is not associated in our sample with differences in square root tooth area, we are confident that variation in relative intercusp distance, measured by a ratio using this size variable in the denominator, along an axis of Carabelli expression will be driven primarily by differences in intercusp distance. Intercusp spacing, as we have measured it at the main cusp tips, cannot be autocorrelated with Carabelli development however assessed because Carabelli trait forms lingual to the main cusps and, in most individuals, further toward the crown case than the main cusps. Because the spacing among the main cusps is determined during dental morphogenesis prior to the initiation of Carabelli trait and other cingular features low on the tooth crown, it is unlikely that the presence of a Carabelli could influence the spacing among the main cusps. Rather, the reverse is far more likely that intercusp distance influences Carabelli expression.

**Table 2:** Comparisons of Mean Intercusp Distance (mm) and Square Root Tooth Area (mm) in left teeth at the extremes of Carabelli development, either Carabelli absent (equivalent to ASU #0) or a fully present Carabelli cusp (equivalent to ASU #5–7).

Comparison	Class	N	Mean	SD	t^1^	p	df
Mean Intercusp Distance	Absent	19	6.302	0.424	3.124***	0.003	74
	Present	57	5.975	0.385			
Square Root Tooth Area	Absent	19	9.754	0.444	−1.521n.s.	0.132	74
	Present	57	9.913	0.378			

^1^t-test for difference between the means of two samples of unequal size and equal variance. For mean intercusp distance, the ratio of the two sample variances, Fs, is 1.213n.s., p = 0.28, and for square root tooth area, Fs = 1.380n.s., p = 0.18, justifying the assumption of equal variance.

**Figure 3:**
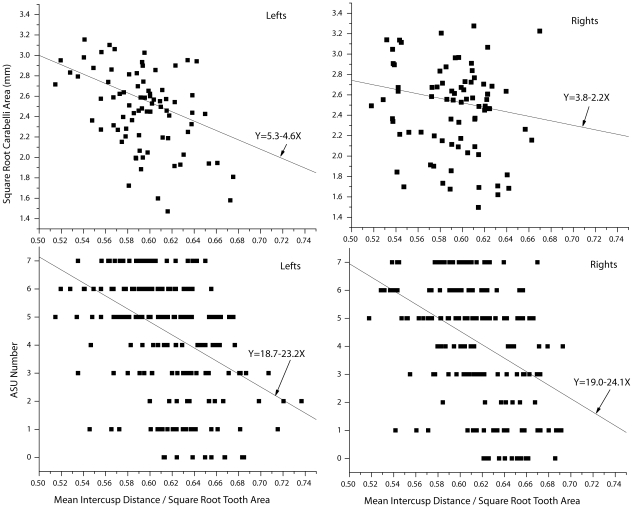
Carabelli expression and relative cusp spacing across individuals (lefts and rights separate).

We analyzed Carabelli expression as assessed through the standardized Arizona State University (ASU) scheme [15] on a scale of 0–7 in two ways, first as a continuous variable and second as an ordered categorical variable. Because designations in the ASU system represent arbitrary points along what is, in nature, a continuum of Carabelli expression, ASU code is not a meristic variable and can be analyzed as a continuous variable [16]. Although Model II regression would be appropriate for determining the functional relationship of Carabelli expression with relative intercusp distance, we instead employ a nonparametric regression alternative using Kendall's robust line-fit method [16], which is less sensitive to the effects of outliers than parametric regression techniques and for which Kendall's rank correlation is an appropriate significance test [16]. Score using the ASU system is negatively correlated with relative intercusp distance for both lefts (Kendall's τ = −0.33, p<0.001, df = 187) and rights (Kendall's τ = −0.32, p<0.001, df = 185; see Figure 3). Across the full range of Carabelli development from none (0) to fully developed cusp (7), teeth with small relative intercusp distances are more likely to develop a Carabelli cusp.

Viewing ASU score as an ordered categorical variable, we estimated the functional relationship of Carabelli expression with relative intercusp distance by proportional odds logistic regression. In this approach, we modeled Carabelli expression as a function of relative intercusp distance as a continuous liability either with seven thresholds, yielding eight observable Carabelli states (ASU codes 0–7), or with two thresholds, yielding three observable Carabelli states (Absent, Slight, Present). Ordered logistic regression, assuming a common slope for the threshold transitions from one state to another, yields one slope coefficient for the whole model and separate intercepts for each threshold. Significance of the whole model is assessed with a likelihood ratio test in comparison with a null model. Results, reported in Table 3, indicate that significant negative relationships exist between these ordinal assessments of Carabelli expression and relative intercusp distance. Because logistic regression coefficients are log-odds ratios, they may be exponentiated to yield the odds ratios of different outcomes in the dependent variable, here degree of Carabelli expression. Scaling the logistic regression coefficients by 0.1 or about one-half of the observed range in relative intercusp cusp distance (∼0.20 or 0.50–0.70) and exponentiating revealed that teeth with comparatively low relative intercusp distances are about four times as likely to have higher Carabelli scores in the simplified three-state system than teeth with 0.1 larger relative intercusp distances. Similarly, teeth with comparatively low relative intercusp distances are eight times more likely to have higher ASU Carabelli scores than teeth with 0.1 higher relative intercusp distances.

**Table 3:** Proportional Odds Logistic Regression of Carabelli expression, Assessed Through Two Typological Schemes and Treated as an Ordered Categorical Variable.

Comparison	Coefficient	Intercepts	LL(H1)^1^	LL(H0)^2^	G^3^
left Absent/Slight/Present	−14.0	−11.2	−162.4	−170.1	15.4***
		−8.0			
right Absent/Slight/Present	−14.5	−11.2	−163.3	−170.4	14.1***
		−8.1			
left ASU#	−20.2	−15.5	−359.0	−378.5	38.9***
		−14.2			
		−13.8			
		−13.1			
		−12.6			
		−11.6			
		−10.3			
right ASU#	−20.6	−15.4	−351.4	−369.2	35.6***
		−14.2			
		−13.9			
		−13.2			
		−12.7			
		−11.7			
		−10.6			

1 LL(H1) = log-likelihood of estimated model.

2 LL(H0) = log-likelihood of null model.

3 G = likelihood ratio or −2(LL(H0)-LL(H1)), in each case greater than the critical value of Chi-square at 1 degree of freedom for p = 0.001, which is 10.828.

We also tested whether Carabelli size itself is correlated with relative intercusp distance in a subsample of teeth in which Carabelli area could be measured, 80 left M1s and 79 right M1s. These teeth included some but not all of those classified with slight or fully developed Carabelli (simplified coding scheme) or ASU numbers 5–7. For left M1s, square root Carabelli cusp area is strongly negatively related to relative intercusp distance (Kendall's τ = −0.26, p<0.001, df = 78; see Figure 3), suggesting that differences in cusp spacing can effect subtle changes in accessory cusp size. For rights, there is considerably more scatter than with lefts (Kendall's τ = −0.096, p = 0.11, df = 77; see Figure 3), and the relation, while not significant, is in the predicted direction. It is unclear why the results from the two sides differed, but we suspect that random measurement error is responsible for obscuring the strength of the negative correlation among the right M1s. There is no biological reason to suspect directional asymmetry in the relation between cusp spacing and cusp size. Nor is there any methodological reason to suspect a systematic bias affecting the measurements of right teeth more than left teeth. Work is underway to investigate this discrepancy between lefts and rights further in an even larger sample of teeth.

**Figure 4:**
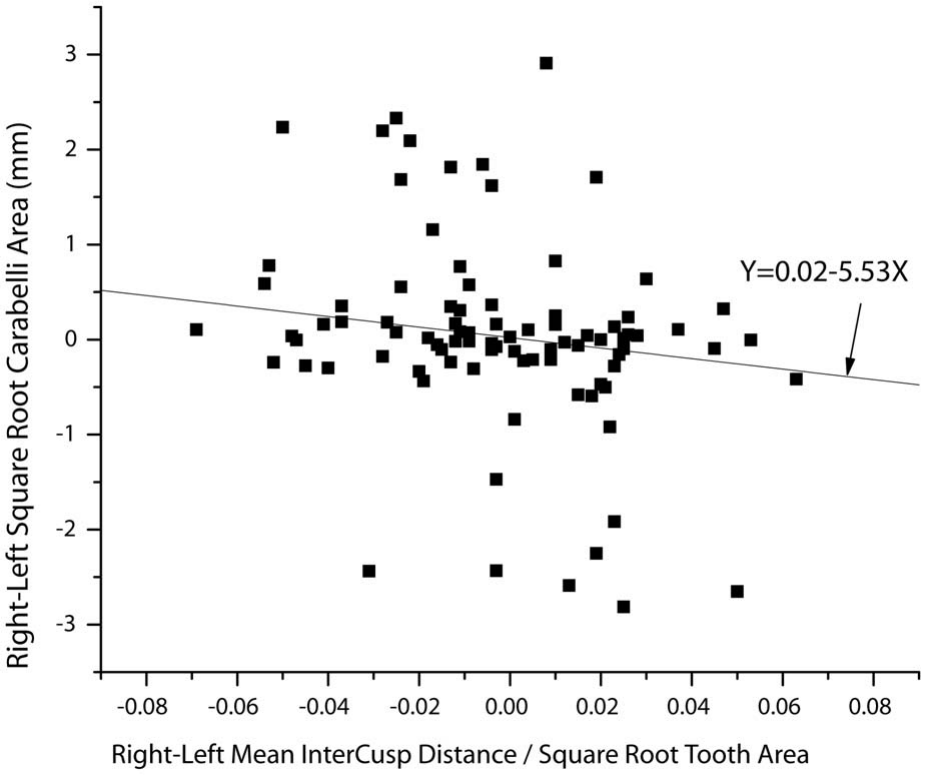
Asymmetry within individuals in Carabelli size and relative cusp spacing.

Finally, for the 89 individuals in which Carabelli area on either left or right teeth could be measured, we tested the hypothesis that individuals with smaller relative intercusp distances on one side would also have larger Carabelli cusps on that side. Figure 4 shows the relation between the right–left differences in square root Carabelli area with right–left differences in relative intercusp distance. As predicted, the two variables are negatively related (Kendall's τ = −0.196, p = 0.003, df = 87). To remove the potential effect of zeros on the correlation (i.e., instances in which we could only measure Carabelli area on right or left teeth, but not both), we also limited the analysis to just the 71 individuals with measureable Carabelli cusp on both sides. In that reduced sample, the negative relation, while still evident, was reduced somewhat in strength and its significance reduced from strongly significant to marginal, but still in the predicted direction (Y = −0.0082–1.8968X; Kendall's τ = −0.1147, p = 0.0793, df = 69). The negative relation between Carabelli size and relative intercusp distance, strongly evident across individuals, is also evident between right and left teeth of the same individuals although the strength of that association, and our ability to detect it, depended on the size and composition of the sample.

## Discussion

Our findings lend support to the patterning cascade model of tooth morphogenesis by validating its main predictions for Carabelli expression: Carabelli is more likely to be present and, if present, is more likely to be large in teeth with low intercusp distances relative to tooth size. Small intercusp distances relative to tooth size implies closely spaced enamel knots relative to the size attained by the tooth by the time that morphogenesis ceases. Such conditions provide greater opportunity for a new enamel knot to form around the periphery of a developing tooth beyond the inhibition fields surrounding earlier-forming enamel knots. Observed negative correlation between Carabelli expression and relative intercusp distance is evident across individuals and extends to right–left differences within the same individual. A common pattern observed both across and within individuals implies that the same underlying factors influence Carabelli expression both within and across individuals and that these factors are not strictly genetic. Small differences in the spacing of enamel knots and the timing of the cessation of morphogenesis most likely determine the probability of forming a new cusp both within and across individuals. High asymmetry in intercuspal differences in modern humans is also consistent with the model because it suggests environmental influences on the formation of enamel knots and the subsequent folding of the enamel epithelium [17].

Jernvall and Jung [11] originally suggested that the patterning cascade model might apply to the formation of a Carabelli cusp, involving the first-formed paracone, and successively formed protocone and Carabelli cusp. Because the cusp form of the Carabelli trait is present at the enamel-dentine junction in human teeth [18], [19], the trait is related to the folding of the enamel epithelium and thus subject to the mechanisms involved in the model. Both Kondo and Townsend [20] and Harris [21] found strong associations between various measures of tooth size (including absolute intercusp distances) and Carabelli expression. These studies are broadly consistent with the patterning cascade model as larger teeth provide additional room for the formation of an enamel knot at the site of the Carabelli cusp. However, the model also predicts that initial spacing of enamel knots can affect whether additional enamel knots form. Holding tooth size constant, additional enamel knots are more likely to form when earlier-forming enamel knots are closely rather than widely spaced. This study investigates the effect of the interaction of cusp spacing with tooth size on Carabelli presence and expression, providing a rigorous test of the model.

Previous researchers of human dental morphology have noted consistency between their data and the broad implications of the patterning cascade model. Townsend et al. [17] found that distances between cusps had larger coefficients of variation and fluctuating asymmetry scores than did measurements of overall crown size. As the authors note, this result supports experimental work indicating that distances between enamel knots, and hence cusp tips, are not under direct genetic control but are the result of “a cascade of epigenetic events” (2003:355). With respect to the Carabelli cusp, there are several studies in which positive associations between cusp presence/expression and crown size have been reported [20], [21], [22], [23], [24]. Kondo and Townsend (2006) suggest that the Carabelli trait will be expressed in its cuspal form if crown growth is prolonged, resulting in both a larger crown and sufficient time for the dental epithelium to fold.

Based on our findings, we believe that it is the interaction between enamel knot spacing and the duration of crown morphogenesis that best reflects the developmental events promoting late-forming enamel knot formation and thus Carabelli cusp expression. Once formed, Carabelli cusp size would be increased either by an earlier onset of formation (resulting in a taller Carabelli cusp), by delaying the cessation of tooth crown formation (resulting in a broader Carabelli cusp, especially lingually), or both. We suspect that pushing the onset of Carabelli formation earlier in development would be promoted more by small distances between early forming enamel knots, whereas broadening the Carabelli would be more strongly promoted by delaying the cessation of growth. In future studies, we hope to tease apart these two very different modes of formation and growth and their relative importance in determining tooth morphogenesis in humans and other mammals.

Carabelli trait has figured prominently in the literature of human dental morphology. Our findings shed light on the phenotypic pattern of Carabelli trait expression. Carabelli, like many features of dental morphology exhibit a “quasi-continuous” mode of variation [25] or “threshold dichotomy” [26]. Such traits do not form below a “physiological threshold” [24] or “phenotypic realization threshold” [27] but vary continuously along a range of expression once the threshold is exceeded. Our data suggest that the patterning cascade model is the developmental mechanism that underlies the observed phenotypic expression of Carabelli. Asymmetry in grades of Carabelli trait expression [28] and intercusp distance, each observed separately, align well with the expectations of the patterning cascade model. Although many studies report no sex difference in Carabelli trait expression, others have found that males are more likely to express the Carabelli cusp than females [20], [29]. A male-bias in Carabelli trait expression is expected given that sex differences in crown size are greater than sex differences in intercuspal distances [17]. Finally, the Carabelli trait is positively associated with the protostylid [30] and the hypocone [31]. Given that these structures are all located peripherally around the main tooth cusps, the conditions that promote the expression of one of them should also promote the expression of the others.

As anthropological studies have used dental morphological traits, including the Carabelli trait, in assessing trends in human populations, interesting geographic and temporal patterns have emerged. Brabant [29] reported an increase in Carabelli trait expression from Neolithic to modern times in Europe, whereas Hsu et al. [32] documented a decrease in Carabelli expression with overall dental reduction from aboriginal to modern Chinese populations. Still others have argued that the trait has decreased in frequency and level of expression over longer evolutionary time scales [31], [33]. Paleoanthropologists have also used characters based on Carabelli expression in phylogenetic analyses of hominin species. Historical and evolutionary patterns of the waxing and waning of Carabelli trait through time and across populations of humans and our closest relatives might be elucidated by our approach. Carabelli trait presence/absence and expression is most meaningfully viewed in the context of variation among other dental characters with which Carabelli trait is phenotypically correlated. We here present evidence that this observed phenotypic correlation between Carabelli trait and other dental features arises out of a developmental correlation. Expression of Carabelli trait and other peripheral features depends on small variations in the relative spacing of earlier-forming enamel knots, the inhibition fields surrounding these enamel knots, and the growth parameters that determine the offset of morphogenesis. In this scheme in which Carabelli expression is determined by upstream events in a developmental cascade, the probability of homoplasy in the expression of Carabelli trait and correlations of Carabelli trait with other dental traits are expected to be high. On the one hand, high probability of homoplasy in Carabelli and strong developmental correlations between Carabelli and other dental traits may present difficulties for phylogenetic analyses that assume character independence [34]. The realization that Carabelli trait and other dental features covary, probably coevolve, and have done so repeatedly alerts us to the chance of being misled by these characters if they are not scored with great care. On the other hand, understanding the developmental basis for Carabelli expression and other dental traits may make it possible to predict with some accuracy patterns of phenotypic expression and coexpression of suites of dental characters that are likely to arise in evolution [35]. Such knowledge of the ontogeny of dental features may make it possible to code as characters developmentally significant events rather than atomized dental traits. In addition, knowledge of favored developmental outcomes might be incorporated as transformation probabilities into the tree-building process itself.

The significance of Carabelli cusp for evolutionary biology is that it provides a well-documented glimpse at the origin of a new cusp. Over the evolutionary history of mammals, which has experienced a large-scale increase in dental complexity, major dental traits have evolved that began at early evolutionary stages as peripheral features, low on a tooth crown, and developed presumably late in ontogeny. One example, the upper molar hypocone, convergently arose in many mammalian groups and transformed in some groups into a main cusp approaching the other main cusps (protocone, paracone, and metacone) in size [36]. The protocone, another example, may have evolved twice during the Mesozoic resulting in tribosphenic molars in both northern-continent tribosphenidans and southern-continent ausktribosphenidans [37]. Transforming a small, low peripheral cusp into a centrally located, large cusp must be accomplished by shifting the initiation of that cusp earlier in ontogeny. Natural selection might drive this heterochronic shift if cusp enlargement results in a new, functionally significant contact with occluding teeth. However, origin of a new cusp in the first place, to use Carabelli expression as a model, can occur as a byproduct of natural variation in the spacing of enamel knots and offset of morphogenesis, which impacts intercusp spacing and tooth size. It is rarely possible to study population-level variation in the early evolutionary stages of the origin of a new cusp in extinct species. Instead, we rely on analyses, such as this one, of variation in small dental features in living species to provide insight in to the origin and evolution of new dental features in the past.

## Materials and Methods

The sample consisted of 376 M1s including 187 rights, 189 lefts, and 185 right–left pairs, in a dental cast sample of Ohio orthodontic patients housed at the Bioarcheology Laboratory of the OSU Department of Anthropology. We measured tooth areas, intercusp distances, and Carabelli cusp areas in two dimensions as projected into the occlusal plane using a Hirox digital microscope at 6× magnification. Teeth were oriented by eye so that the widest part of the crown was horizontally level. We measured areas of teeth and Carabelli cusp as the areas enclosed within a set of 20–30 points surrounding either the entire tooth or Carabelli cusp, respectively. We were able to measure Carabelli area in 89 individual dental casts on either the right side (79) or the left side (80). In the entire sample 376 M1s, we scored Carabelli development using two typological schemes. We employed a simplified scheme labeling Carabelli as “present” where Carabelli area was measurable, “slight” where Carabelli development was evident but not measurable (i.e., not clearly separable from the protocone), or “absent” where Carabelli cusp was not evident whatsoever. We also employed the standardized Arizona State University dental plaque scheme [15] coding Carabelli development on a scale from 0 (absent) through 7 (fully independent Carabelli cusp).
